# Molecular Evolution of MERS Coronavirus: Dromedaries as a Recent Intermediate Host or Long-Time Animal Reservoir?

**DOI:** 10.3390/ijms18102138

**Published:** 2017-10-16

**Authors:** Susanna K. P. Lau, Antonio C. P. Wong, Terrence C. K. Lau, Patrick C. Y. Woo

**Affiliations:** 1 State Key Laboratory of Emerging Infectious Diseases, The University of Hong Kong, Pokfulam, Hong Kong; 2 Department of Microbiology, Li Ka Shing Faculty of Medicine, The University of Hong Kong, Pokfulam, Hong Kong; antonwcp@hku.hk; 3 Research Centre of Infection and Immunology, the University of Hong Kong, Pokfulam, Hong Kong; 4 Carol Yu Centre for Infection, the University of Hong Kong, Pokfulam, Hong Kong; 5 Collaborative Innovation Centre for Diagnosis and Treatment of Infectious Diseases, the University of Hong Kong, Pokfulam, Hong Kong; 6 Department of Biomedical Sciences, College of Science and Engineering, City University of Hong Kong, Tat Chee Avenue, Kowloon, Hong Kong; chiklau@cityu.edu.hk

**Keywords:** MERS coronavirus, molecular, evolution, dromedaries, origin, recent, host, reservoir

## Abstract

While dromedary camels are the immediate animal source of MERS coronavirus (MERS-CoV) infection, the evolutionary origin of MERS-CoV remains obscure. We analyzed 219 camel and human MERS-CoV genome sequences available in GenBank. Phylogenetic analysis showed that 5 and 214 strains belong to clade A and B, respectively, with clade A further divided into lineage A1 (3 human strains) and lineage A2 (2 camel strains), and clade B divided into B1 to B6 (each containing both human and camel strains). Recombination analysis showed potential recombination events in five strains from dromedaries in Saudi Arabia, with recombination between lineage B5 and B3 in four strains, and between lineage B3 and B4 in one strain. The spike protein showed the highest number of amino acid substitutions, especially between A2 and other lineages, and contained positively selected codons. Notably, codon 1020 was positively selected among B and B5 strains, and can distinguish between clade A (Q1020) and B (R1020/H1020) strains, suggesting that this residue may play a role in the evolution of S protein during divergence of different lineages. The time of the most recent common ancestor of all MERS-CoV was dated to approximately 2010. The implications on the role of camels in the evolution of MERS-CoV are discussed.

## 1. Introduction

Since its first appearance in 2012, the Middle East Respiratory Syndrome (MERS) has affected more than 25 countries in four continents with more than 1900 cases and a frightening fatality rate of more than 30% [1]. A novel lineage C betacoronavirus, MERS coronavirus (MERS-CoV), has been confirmed to be the etiological agent of MERS [2,3]. Subsequent detection of MERS-CoV and its antibodies in dromedaries in various countries in the Middle East and North Africa have implied that these animals are probably the reservoir for MERS-CoV [4,5,6]. In addition, the discovery of other closely related lineage C betacoronaviruses both before and after the MERS epidemic in various bat species and hedgehogs [7,8,9,10], as well as the demonstration of the ability of the spike protein of *Tylonecteris* bat CoV HKU4 to bind dipeptidyl peptidase 4 [11,12], the receptor of MERS-CoV, have also suggested that these animals could be the hosts for the ancestor of MERS-CoV. However, it is still uncertain how and when the ancestor of MERS-CoV may have jumped from bats/hedgehogs to camels and humans. 

Up to March 2017, a total of more than 200 complete genome sequences of MERS-CoV are available in GenBank, with around half of them from MERS-CoVs found in human and the other half from MERS-CoVs in dromedaries. Based on our previous studies on genome sequencing and phylogenetic analysis of MERS-CoVs from dromedaries of the United Arab Emirates (UAE), we observed that diverse MERS-CoV strains were circulating in these dromedaries, supporting a polyphyletic origin [13]. In this study, we analyzed the diversity and phylogeny of all the 219 human and dromedary MERS-CoV genome sequences in GenBank. Possible recombination events among the MERS-CoV strains were also detected. The implications of the results on the origin and control of MERS, and the role of dromedary camels in the evolution of MERS-CoV, are discussed. 

## 2. Results

### 2.1. Human and Dromedary MERS-CoV Genomes

**Figure 1:**
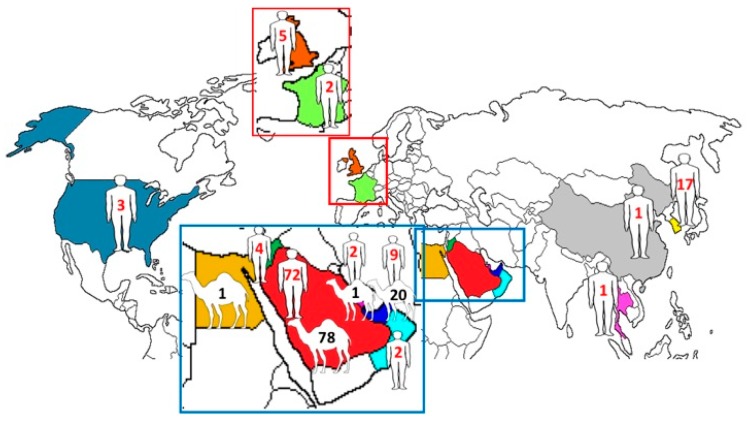
Geographical distribution of human and dromedary MERS-CoV strains with complete genomes. The numbers represent the number of MERS-CoV strains with complete genomes reported from the corresponding countries.

Among the 219 complete genomes of MERS-CoV, 151 were from Saudi Arabia (human, *n* = 73; dromedary, *n* = 78), 29 were from the UAE (human, *n* = 9; dromedary, *n* = 20), 4 were from Jordan (human, *n* = 4), 1 was from Egypt (dromedary, *n* = 1), 5 were from the United Kingdom (human, *n* = 5), 3 were from Qatar (human, *n* = 2; dromedary, *n* = 1), 3 were from the United States (human, *n* = 3), 2 were from France (human, *n* = 2), 2 were from Oman (human, *n* = 2), 1 was from Thailand (human, *n* = 1), 1 was from China (human, *n* = 1), and 17 were from South Korea (human, *n* = 17) (Figure 1).

### 2.2. Phylogenetic Analysis and Genomic Sequence Comparison

**Figure 2:**
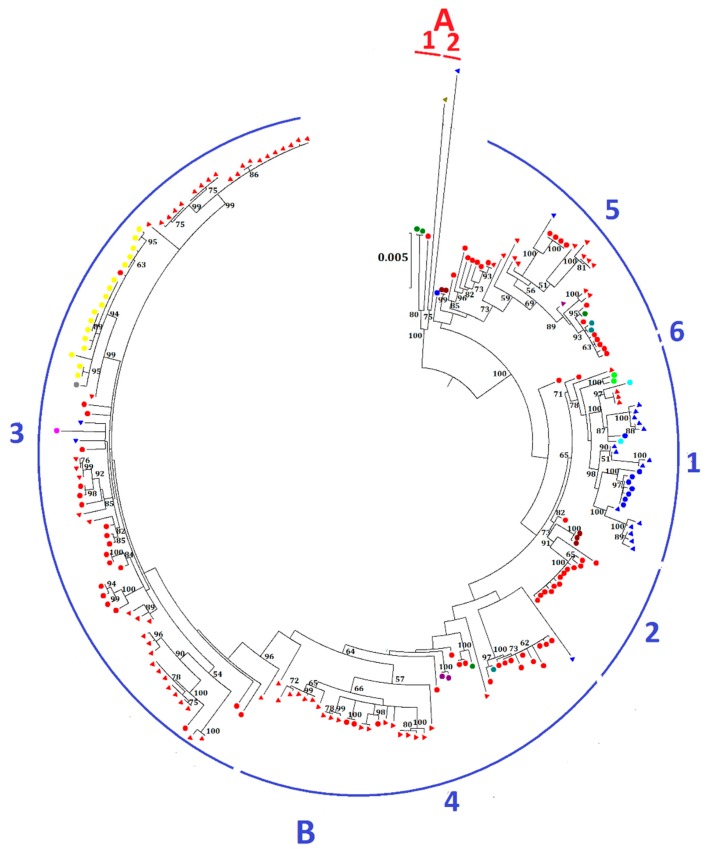
Maximum-likelihood phylogeny based on the complete genome sequences of 219 MERS-CoV strains. A general time-reversible model of nucleotide substitution with estimated base frequencies, the proportion of invariant sites, and the γ distribution of rates across sites were used in the maximum-likelihood analysis. Bootstrap values in percentage are shown next to the branches. The scale bar indicates the number of nucleotide substitutions per site. Different colored letters and numbers represent different clades and lineages respectively: red letter, clade A; red number, lineages within clade A; blue letter, clade B; blue numbers, lineages within clade B. MERS-CoVs from dromedaries and humans are indicated in triangles and circles, respectively. Different colors represent MERS-CoV strains found in different countries: red, Saudi Arabia; blue, United Arab Emirates; green, Jordan; olive, Egypt; maroon, United Kingdom; purple, Qatar; teal, United States; lime, France; aqua, Oman; fuchisa, Thailand; grey, China; yellow, South Korea.

The 219 complete genomes of MERS-CoV were clustered into two major clades, A and B (Figure 2). Most MERS-CoV strains belong to clade B, while clade A only contains five strains. Clade A is further divided into two lineages: lineage A1, with three human strains, one being the first MERS-CoV strain isolated from the index 60-year-old male patient from Bisha, Saudi Arabia in September 2012, and the other two from Jordan; and lineage A2, with two camel strains, one from the UAE and the other from Egypt. Clade B is further divided into six lineages. Both human and camel strains were present in most lineages. Lineage B1 is dominated by MERS-CoV strains from the UAE, whereas lineages B2 to B6 are mainly dominated by strains from Saudi Arabia. Lineage 3 consists of the largest number of strains, including the human strains from an outbreak in South Korea, which occurred as a result of acquisition of MERS-CoV by a Korean who visited multiple Middle East countries, including Bahrain, Qatar, Saudi Arabia, and the UAE from 24 April to 4 May 2015 and subsequent spread the infection after returning to South Korea [14]. The same strain of MERS-CoV was also carried to China by a 44-year-old male Korean [15]. This patient visited his father and sister, who were infected with MERS-CoV in South Korea, before traveling to Hong Kong on 26 May and subsequently to Huizhou in the Guangdong province through Shenzhen City. 

**Figure 3:**
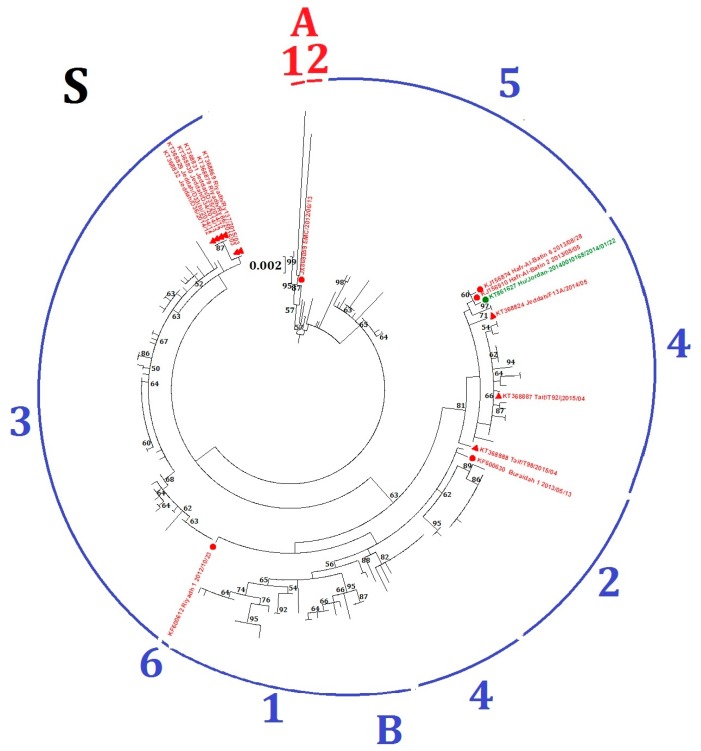
Maximum-likelihood phylogeny based on *S* gene sequences of 219 MERS-CoV strains. GTR + G + I substitution model was selected for the *S* gene tree. Bootstrap values in percentage are shown next to the branches. The scale bar indicates the number of nucleotide substitutions per site. Different colored letters and numbers represent different clades and lineages respectively: red letter, clade A; red number, lineages within clade A; blue letter, clade B; blue numbers, lineages within clade B. Selected MERS-CoV strains were annotated. MERS-CoVs from dromedaries are indicated in triangles. MERS-CoVs from homo sapiens are indicated in circles. The 15 MERS-CoV strains with potential recombination detected in the present study are colored: red, Saudi Arabia; green, Jordan.

Fifteen MERS-CoV strains, including six from human and nine from dromedaries, fell into different lineages in the phylogenetic trees constructed using ORF1ab and *S* (Table 1, Figure S1A and Figure 3), suggesting possible recombination events. For example, four MERS-CoV strains from dromedaries (accession numbers KT368829, KT368830, KT368831, KT368832) belong to lineage B5 in the ORF1ab tree but lineage B3 in the *S* tree; and three MERS-CoV strains from human (accession numbers KT861627, KJ156910, KJ156874) belong to lineage B2 in the ORF1ab tree but lineage B4 in the *S* tree.

**Figure 4:**
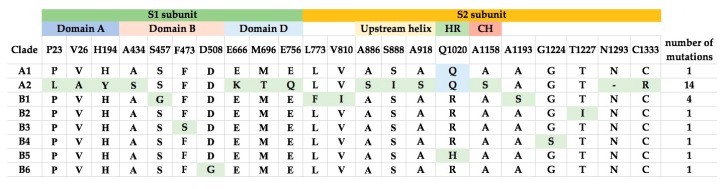
Amino acid substitutions in S proteins from the different clades of MERS-CoV. The S1 and S2 subunits, and structural domains are highlighted. The amino acid substitutions are highlighted and the total number of mutations in each clade are shown in the last column.

Comparison of the amino acid sequences of the various lineages of MERS-CoV revealed that the number of changes in the S proteins is higher than in other proteins. Notably, MERS-CoV of lineage A2 showed 14 amino acid changes compared to other lineages, while most other lineages (A1, B2–6) only showed one amino acid change compared to other lineages (Figure 4). These amino acid changes are not concentrated in particular motifs (Figure 4).

### 2.3. Recombination Analysis

**Figure 5:**
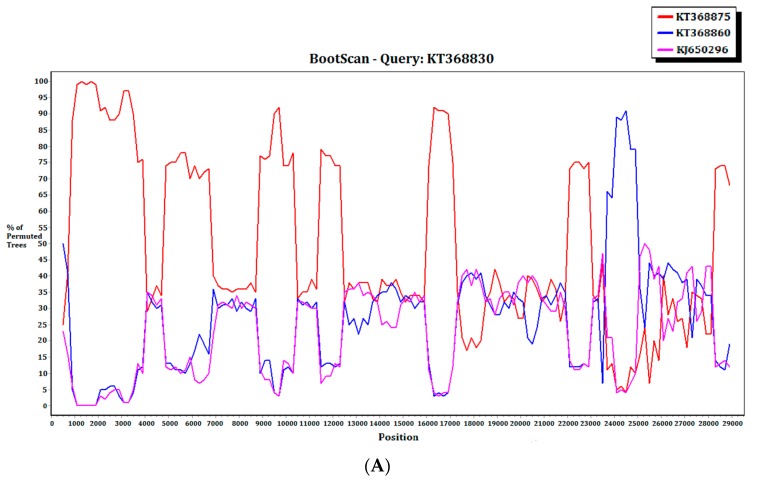

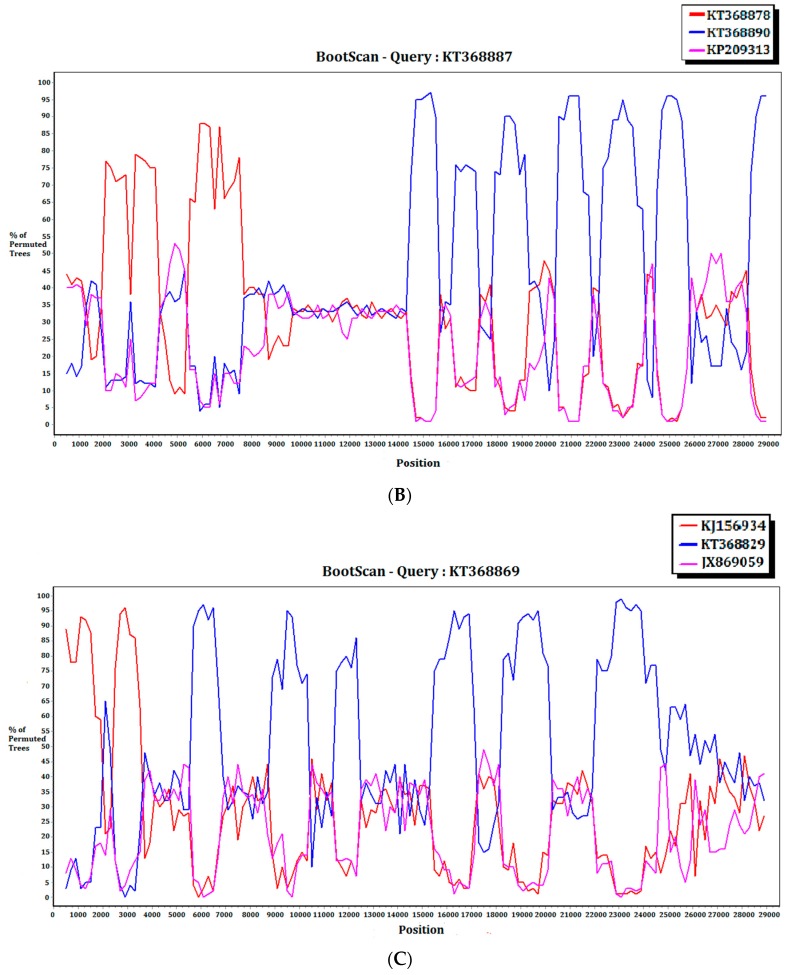

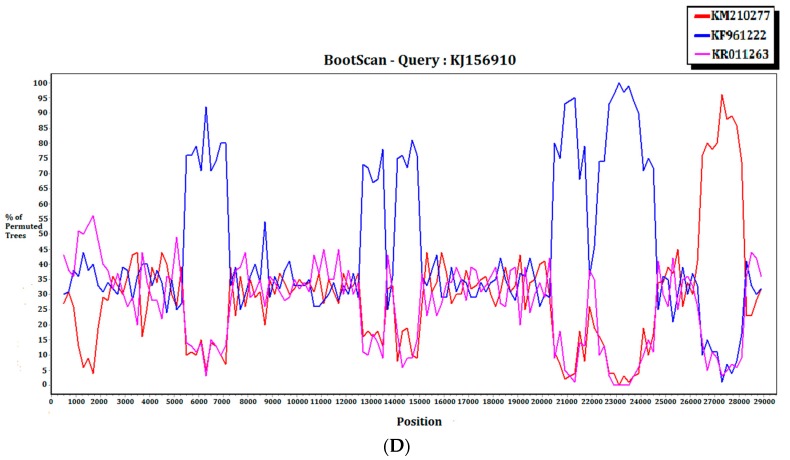
Detection of potential recombination events by bootscan analysis. Bootscanning was conducted with Simplot version 3.5.1 (F84 model; window size, 1000 bp; step, 200 bp). (**A**) KT368830 was used as the query sequence and compared with the genome sequences of a lineage B5 MERS-CoV strain (red, KT368875), a lineage B3 MERS-CoV (blue, KT368860), and a lineage B1 MERS-CoV strain (purple, KJ650926); (**B**) KT368887 was used as the query sequence and compared with the genome sequences of a lineage B3 MERS-CoV strain (red, KT368878), a lineage B4 MERS-CoV strain (blue, KT368890), and a lineage B1 MERS-CoV strain (purple, KP209313); (**C**) KT368869 was used as the query sequence and compared with the genome sequences of a lineage B4 MERS-CoV strain (red, KJ156934), a lineage B3 MERS-CoV (blue, KT368829), and a lineage A1 MERS-CoV strain (purple, JX869059); (**D**) KJ156910 was used as the query sequence and compared with the genome sequences of a lineage B2 MERS-CoV strain (red, KM210277), a lineage B4 MERS-CoV (blue, KF961222), and a lineage B5 MERS-CoV strain (purple, KR011263).

For the 15 MERS-CoV genomes that showed different clustering in the ORF1ab and *S* phylogenetic trees, they were subject to recombination analysis. Among these 15 strains, bootscan analysis revealed possible recombination sites in five (Figure 5A,B). These five strains were all from dromedaries in Saudi Arabia. They were separated into two groups, with one group containing four strains from Jeddah of almost identical sequences (accession Nos. KT368829, KT368830. KT368831, KT368832) and the remaining one strain from Taif (accession No. KT368887). For the other 10 strains, bootscan analysis did not reveal any obvious pattern that suggested possible recombination (Figure 5C,D). 

### 2.4. Multiple Alignment Analysis

When the 15 MERS-CoV genomes that showed different clustering in the ORF1ab and *S* phylogenetic trees were subject to multiple alignment analysis, it was shown that for the five strains with possible recombination revealed by bootscan analysis, the possible recombination sites were confirmed by multiple alignments. For four strains (accession No. KT368829, KT368830, KT368831 and KT368832), sequences upstream to the possible recombination site were similar to that of KT368875 from lineage B5, while those downstream to the possible recombination site were similar to that of KT368860 from lineage B3 (Figure S2A). As for the remaining strain (accession No. KT368887), sequence upstream to the possible recombination site was similar to that of KT368878 from lineage B3, while sequence downstream to the possible recombination site was similar to that of KT368890 from lineage B4 (Figure S2B).

### 2.5. Selective Pressure Analysis

To assess adaptive evolution of MERS-CoV, dN/dS ratios (*ω*) in *S* gene across the 219 strains were calculated on codon-by-codon basis. The overall *ω* was 0.424737 (0.392404 and 0.439492 for clade A and B strains, respectively), with most codons having *ω* < 1, indicating purifying selection (Table 2). Nevertheless, four codons were predicted to have *ω* > 1 by different methods with statistical significance, including codons 26 and 312 by fixed effects likelihood (FEL) method, codon 1250 by mixed-effects model of evolution (MEME) method, and codon 1224 by both FEL and MEME methods, indicating possible functional constraints at these positions during evolution. Among them, codons 1224 and 1250 were also predicted to be positively selected sites among clade B strains by at least one method (Table 2). In addition, codon 879 was predicted to be positively selected among clade A strains, whereas codons 312 and 1020 were predicted to be positively selected among clade B strains by at least one method (Table 2). Furthermore, codons 1250 and 1020 were predicted to be positively selected among clade B3 and B5 strains, respectively, by MEME method (Table 2). However, none of these amino acid residues were within the receptor binding domain of MERS-CoV, suggesting that there has not been strong selective pressure at the receptor-binding interphase during virus evolution.

### 2.6. Modeling of Spike Protein

**Figure 6:**
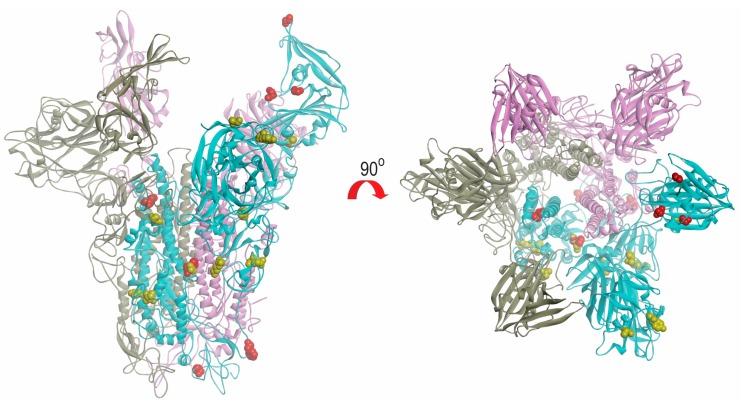
Structural view of amino acid substitutions in S protein of different clades of MERS-CoV. The mutated amino acids in clade A and B are highlighted in yellow and red colour, respectively. The figures were produced using Discovery Studio visualizer (Accelrys).

The models of spike glycoprotein from each clade were built. It was observed that the amino acid substitutions observed by multiple alignments appeared to be randomly distributed throughout the structure (Figure 6). Based on the model, we did not observe specific evolutionary patterns of the MERS-CoV spike protein. However, intriguingly, the residue at position 1020 can distinguish between clade A and B (Figure 4). The glutamine (Q1020) and arginine residues (R1020) at that position are highly conserved in clade A and clade B, respectively, except for a histidine residue (H1020) found in 12 lineage B5 strains. Together with results from selective pressure analysis, this suggests that this amino acid residue may play a role during the molecular evolution of S protein of MERS-CoV and divergence of different lineages.

### 2.7. Estimation of Divergence Dates

**Figure 7:**
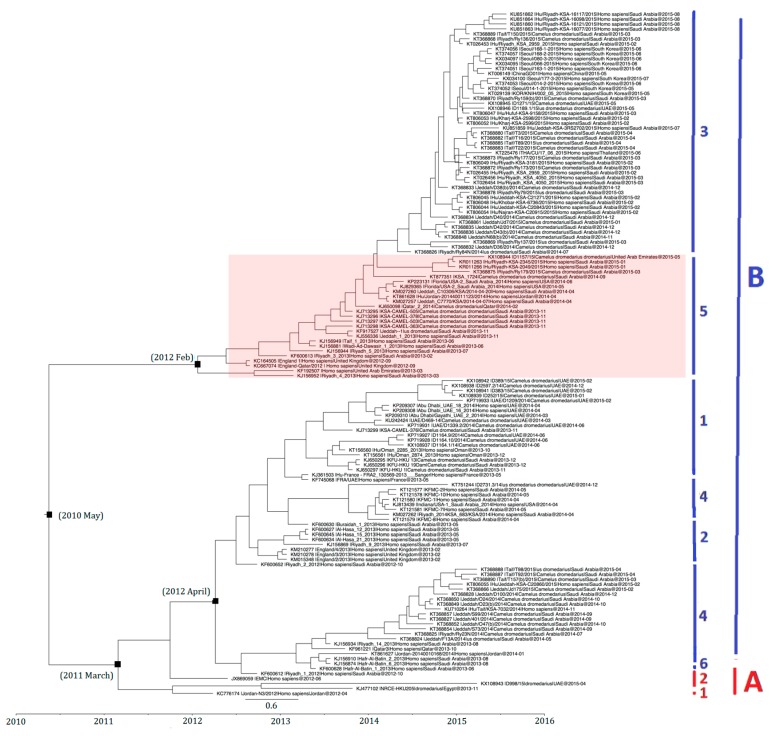
Estimation of time to the most recent common ancestor for MERS-CoV. The time-scaled phylogeny was summarized from all Markov chain Monte Carlo phylogenies of the concatenated coding region S of 138 phylogenetically distinct MERS-CoV genomes, which were analyzed under the relaxed-clock model with an uncorrelated exponential distribution in BEAST version 1.7.4. The clade B lineage 5 MERS-CoV is highlighted. Different colored letters and numbers represent different clades and lineages respectively: red letter, clade A; red number, lineages within clade A; blue letter, clade B; blue numbers, lineages within clade B.

The divergence time of 138 selected MERS-CoV strains based on *S* gene analysis is shown in Figure 7. Strains with similar sequences or collection dates were excluded. For example, only 9 out of 17 strains from South Korea were included. On the basis of time-resolved phylogeny, the root of the tree was dated to May 2010 (95% HPD: August 2007, October 2011). The time of the most recent common ancestor (tMRCA) of clade A was dated back to March 2011 (95% HPD: December 2009, December 2011) and that of clade B lineages 3 and 5 to February 2012 (95% HPD: April 2011, July 2012), while clade B lineages 1, 2, 4, and 6 to April 2012 (95% HPD: September 2011, August 2012) (Figure 7). 

## 3. Discussion

Among all known CoVs, the largest numbers of complete/near-complete genomes are available for SARS-CoV and MERS-CoV, of which >300 and >200 genomes, respectively, are available for analysis. Such large number of genomes has given us a good opportunity to improve our understanding on the origin and evolution of the corresponding viruses. In contrast to the major SARS outbreak in 2003 which is of monophyletic origin, MERS-CoVs circulating in human and camels since 2012 originated from multiple sources in the Middle East and North Africa. For example, strains from a single country such as the UAE are present in lineages A2, B1, 3, 4, and 5, and strains from Jordan are also present in lineages A1, B4, and B5. This indicated that different MERS-CoV strains have resided in dromedaries in different countries with sporadic transmission to humans, sometimes causing fatal diseases. Overall, some minor recombination events between different sublineages (between B5 and B3, and between B3 and B4) might have occurred (Figure 5 and Figure 6), but it seems that none of these events has resulted in significant changes in the genome. This is in contrast to some CoVs such as human CoV HKU1, human CoV OC43, or canine CoVs where recombination events are responsible for the generation of novel genotypes [16,17,18]. 

While there is evidence suggesting that MERS-CoV may have infected camels for at least a few decades, it is intriguing that the present molecular clock analysis using available dated MERS-CoV genome sequences suggests a relatively recent common ancestor dated to approximately 2010. Based on existing evidence, there are two possibilities in the evolutionary pathway of MERS-CoV. The first possibility is that MERS-CoV has resided in camels for long time before infecting humans to cause the epidemic in 2012. The alternative hypothesis is that MERS-CoV has only emerged in camels recently from an unknown animal reservoir, and was spilled over to humans because of efficient receptor binding to human dipeptidyl peptidase 4 when a sufficient proportion of camel population was infected. The first hypothesis is supported by two previous serological studies showing that MERS-CoV neutralizing antibodies in archived sera samples of dromedaries in Eastern Africa and Kenya can be detected as early as 1980s and 1990s [19,20]. Moreover, the lack of positive selection at the receptor binding domain of spike protein of MERS-CoV may suggest the virus has been well adapted in camels and humans for a period of time. However, several questions were not readily answered by this hypothesis. First, since genomes of MERS-CoVs from humans and camels were intermingled and closely related without major adaptation before transmission to humans, one would expect similar camel-to-human transmission efficiency before the epidemic in 2012. However, no human cases before 2012 were reported so far, and more importantly, MERS-CoV has not been detected in any archived human or camel samples before 2012. Nevertheless, there is a possibility that early human cases may have been missed if they were not investigated for coronaviruses. Yet, if MERS-CoV has been circulating in camels for long time, one would expect a much earlier estimated tMRCA by molecular clock analysis using available MERS-CoV genome sequences. Although the present study may be limited by the lack of “historical” strains of MERS-CoV for analysis, the diverse circulating strains detected since 2012 should have reflected the history of evolution of MERS-CoV and allow a fair estimation of tMRCA. Nevertheless, isolation of MERS-CoV or detection of viral RNA from archived samples will be important to confirm if camels are indeed long-time reservoir of MERS-CoV.

The evolutions of clade A and clade B MERS-CoV are quite different from each other. Intriguingly, clade B MERS-CoV out-numbered clade A MERS-CoV strains (Figure 2). This may be a result of sampling bias or a genuine survival or transmission advantage of clade B MERS-CoV. As for the specific lineages, the S protein of lineage A2 MERS-CoV showed higher number of amino acid changes as compared to other lineages. After the virus has evolved into clades A and B, we speculate that lineage B5 diverged and evolved separately from the other clade B lineages, as demonstrated by its deep branch in the phylogenetic tree as well as the unique amino acid change at position 1020 of the S2 subunit in S protein (Figure 4 and Figure 6). Moreover, this residue at 1020 also distinguishes between clade A and B strains, and was positively selected among B and B5 strains, suggesting a role in the divergence of different lineages along the evolution of spike protein. Interestingly, this residue is located at the HR region of S2 which is responsible for virus-host fusion. Further studies have to be performed to understand whether clade B MERS-CoV have better survival advantages over clade A MERS-CoV, and the potential effect of amino acid substitution at codon 1020 on viral fusion.

In addition to understanding the evolutionary histories of the corresponding viruses, genome analysis has provided important insights to the approach of controlling the clinical diseases of SARS and MERS. The major SARS outbreak in humans in early 2003 originated from a single or very few civet-to-human interspecies jumping events in Southern China, which explains the monophyletic evolution of strains involved in this outbreak. As a result, the epidemic was successfully controlled when the animal source was segregated from humans after closure of wild life wet markets in Southern China. On the other hand, MERS-CoV had been endemic in dromedary population in the Middle East during the outbreak in 2012 with many independent, sporadic camel-to-human transmission events in Saudi Arabia and the neighboring countries. Since MERS-CoV is found in dromedary calves in most countries in the Middle East and Africa, it would be impossible to control the disease by closure of a limited number of facilities in a restricted geographical region. For visitors to the Middle East, it is important to improve education so that they will avoid contacts with dromedaries, particularly dromedary calves. For residents of the Middle East, more viable options may be through the development of rapid laboratory diagnostics, antivirals, and vaccines for treatment and prevention of MERS. 

## 4. Materials and Methods

### 4.1. Human and Dromedary MERS-CoV Genomes

Up to 10 March 2017, a total of 223 “complete” genome sequences of MERS-CoV were found in GenBank. Four of these 223 MERS-CoV “complete” genome sequences have an incomplete 5′ genomic region or are redundant sequences of a single strain from GenBank, and therefore were not included in the analysis. As a result, 219 complete genome sequences of MERS-CoV, including 119 from MERS-CoV found in humans and 100 from MERS-CoV in dromedaries, were included in the analysis.

### 4.2. Phylogenetic Analysis and Genomic Sequence Comparison

The 219 MERS-CoV genomes were aligned by multiple alignments using fast fourier transform. Maximum-likelihood phylogenetic trees with 1000 bootstrap replicates were constructed using PhyML v3.0 (The French Institute of Bioinformatics & France Genomique, Montpellier, France) on the basis of their complete genomes as well as ORF1ab and *S* genes. The best-fit substitution model was selected using PhyML with Smart Model Selection and used in the maximum-likelihood analysis. 

### 4.3. Recombination Analysis

To detect possible recombination, bootscan analysis with 1000 bootstrap replicates was performed by using the nucleotide alignment of the genome sequences of MERS-CoV and Simplot version 3.5.1 (SCRoftware, USA), as previously described [16,17]. The analysis was conducted using a sliding window of 1000 nucleotides moving in 200 nucleotide steps with genome sequences obtained in the present study as the query. Possible recombination sites suggested by the bootscan analysis were confirmed through multiple sequence alignments. 

### 4.4. Multiple Alignment Analysis

For genomes that showed possible recombination in bootscan analysis, the nucleotide sequences of the corresponding genomes were analyzed by multiple alignment using ClustalW available in BioEdit Sequence Alignment Editor Version 7.2.5 (Ibis Therapeutics, Carlsbad, CA, USA). Nucleotide mutations in the genomes were revealed. 

### 4.5. Selective Pressure Analysis on Spike Protein

The number of synonymous substitutions per synonymous site, dS, and non-synonymous substitutions per non-synonymous site, dN, in S were calculated. Sites under positive selection were inferred using different methods as described in previous publications [21,22,23,24]. First, single-likelihood ancestor counting (SLAC) and fixed effects likelihood (FEL) methods were used as implemented in DataMonkey server (Available online: http://www.datamonkey.org). The overall *ω* (dN/dS) value was calculated according to NJ trees under the custom (010121 with AIC of 16604.47594182065) substitution model. The *ω* (dN/dS) values for different clades and lineages were calculated according to NJ trees under either the F81 model or the custom substitution model (010121, 010011, 001020, or 000010 with various AIC). Positive selection for a site was considered to be statistically significant if *p*-value was <0.1. A mixed-effects model of evolution (MEME) was further used to identify positively selected sites under episodic diversifying selection in particular positions among different clades within a phylogenetic tree, even when positive selection was not evident across the entire tree. 

### 4.6. Modeling of Spike Protein

Models of representative sequences of the S proteins from each clade were built with the structure of MERS-CoV S protein (5X59) using homology modeling (SWISS-MODEL) with default parameters. The models were then superimposed and analyzed using Discovery Studio visualizer (Accelrys) (BIOVIA, San Diego, CA, USA).

### 4.7. Estimation of Divergence Dates

Divergence times for the MERS-CoV strains were calculated using a Bayesian Markov chain Monte Carlo (MCMC) approach implemented in BEAST (version 1.7.4) (BEAST Developers, Open Source, available online: beast.community/programs), as described previously [25,26]. Representative strains were selected for MERS-CoV strains with close sequence similarity and obtained from the same outbreak. Analyses were performed under the SRD06 substitution models for the spike coding regions of the genome (S), with an uncorrelated exponential relaxed clock and a constant size coalescent model. The MCMC run was 5 × 10^7^ steps long with sampling every 1000 steps. Convergence was assessed on the basis of the effective sampling size after a 10% burn-in using Tracer software, version 1.6.0 (Available online: http://tree.bio.ed.ac.uk/software/tracer/). The mean time to tMRCA and the highest posterior density regions at 95% (HPDs) were calculated. The trees were summarized in a target tree by using the Tree Annotator program included in the BEAST package by choosing the tree with the maximum sum of posterior probabilities (maximum clade credibility) after a 10% burn-in. 

## Figures and Tables

**Table 1:** MERS-CoV strains that showed differential clustering for ORF1ab and *S* gene.

GenBank Accession No.	Country of Isolation	Human/Dromedary	Lineage Whole Genome	ORF1ab	*S*
JX869059	Saudi Arabia	Human	A1	A1	A2
KF600630	Saudi Arabia	Human	B1	B1	B2
KT368869	Saudi Arabia	Dromedary	B3	B4	B3
KT368879	Saudi Arabia	Dromedary	B3	B4	B3
KT368831	Saudi Arabia	Dromedary	B5	B5	B3
KT368830	Saudi Arabia	Dromedary	B5	B5	B3
KT368829	Saudi Arabia	Dromedary	B5	B5	B3
KT368832	Saudi Arabia	Dromedary	B5	B5	B3
KT861627	Jordan	Human	B4	B2	B4
KJ156910	Saudi Arabia	Human	B4	B2	B4
KJ156874	Saudi Arabia	Human	B4	B2	B4
KT368888	Saudi Arabia	Dromedary	B3	B3	B4
KT368887	Saudi Arabia	Dromedary	B3	B3	B4
KT368824	Saudi Arabia	Dromedary	B4	B5	B4

**Table 2:** Selective pressure analysis on the *S* genes of 219 MERS-CoV strains.

Clade/Lineage	dN/dS	Positively Selected Sites by SLAC Method	Positively Selected Sites by FEL Method	Positively Selected Sites by MEME Method	Best Fit Model
A and B	0.424737	Nil	Codon 26 *, 312 *, 1224 *	Codon 1224 *, 1250 *	010121
A	0.392404	Nil	Nil	Codon 879 *	010121
B	0.439492	Nil	Codon 312 *, 1224 *	Codon 1020 *, 1224 *, 1250 *	010121
A1	0.234930	Nil	Nil	Nil	F81
A2	Insufficient available strains for analysis				
B1	0.382699	Nil	Nil	Nil	010011
B2	0.405071	Nil	Nil	Nil	010121
B3	0.400104	Nil	Nil	Codon 1250 *	001020
B4	0.641872	Nil	Nil	Nil	000010
B5	0.343586	Nil	Nil	Codon 1020 *	010121
B6	Insufficient available strains for analysis				

SLAC: single-likelihood ancestor counting; FEL: fixed effects likelihood; MEME: mixed-effects model of evolution; * *p* < 0.1.

## Supplementary Materials

Supplementary materials can be found at www.mdpi.com/1422-0067/18/10/2138/s1.

## Abbreviations

AIC	Akaike’s information criterion
BEAST	bayesian evolutionary analysis by sampling trees
dN	the number of non-synonymous substitutions per non-synonymous site
dS	The number of synonymous substitutions per synonymous site
FEL	fixed effects likelihood
HPD	highest posterior density
MCMC	markov chain Monte Carlo
MEME	mixed-effects model of evolution
MERS-CoV	middle east respiratory syndrome coronavirus
ORF	open reading frame
S	spike
SARS-CoV	severe acute respiratory syndrome coronavirus
SLAC	single-likelihood ancestor counting
tMRCA	the most recent common ancestor
