# Supplementary material for: Molecular Evolution of MERS Coronavirus: Dromedaries as a Recent Intermediate Host or Long-Time Animal Reservoir?
Source: Int J Mol Sci. 2017 Oct 16;18(10):2138. doi: 10.3390/ijms18102138 (PMC5666820; doi:10.3390/ijms18102138)
Supplement: Supplementary file 1 [file ijms-18-02138-s001.docx]

Molecular Evolution of MERS Coronavirus: Dromedaries as a Recent Intermediate Host or
Long-Time Animal Reservoir?

Susanna K. P. Lau ^1,2,3,4,5,^*, Antonio C. P. Wong ^2^, Terrence C. K. Lau ^6^ and Patrick C. Y. Woo ^1,2,3,4,5,^*

^1^ State Key Laboratory of Emerging Infectious Diseases, The University of Hong Kong, Pokfulam,
Hong Kong

^2^ Department of Microbiology, Li Ka Shing Faculty of Medicine, The University of Hong Kong, Pokfulam, Hong Kong; antonwcp@hku.hk

^3^ Research Centre of Infection and Immunology, the University of Hong Kong, Pokfulam, Hong Kong

^4^ Carol Yu Centre for Infection, the University of Hong Kong, Pokfulam, Hong Kong

^5^ Collaborative Innovation Centre for Diagnosis and Treatment of Infectious Diseases, the University of Hong Kong, Pokfulam, Hong Kong

^6^ Department of Biomedical Sciences, College of Science and Engineering, City University of Hong Kong, Tat Chee Avenue, Kowloon, Hong Kong; chiklau@cityu.edu.hk

***** Correspondence: skplau@hku.hk (S.K.P.L.); pcywoo@hku.hk (P.C.Y.W.);
Tel.: +852-2255-4892 (S.K.P.L. & P.C.Y.W.); Fax: +852-2855-1241 (S.K.P.L. & P.C.Y.W.)


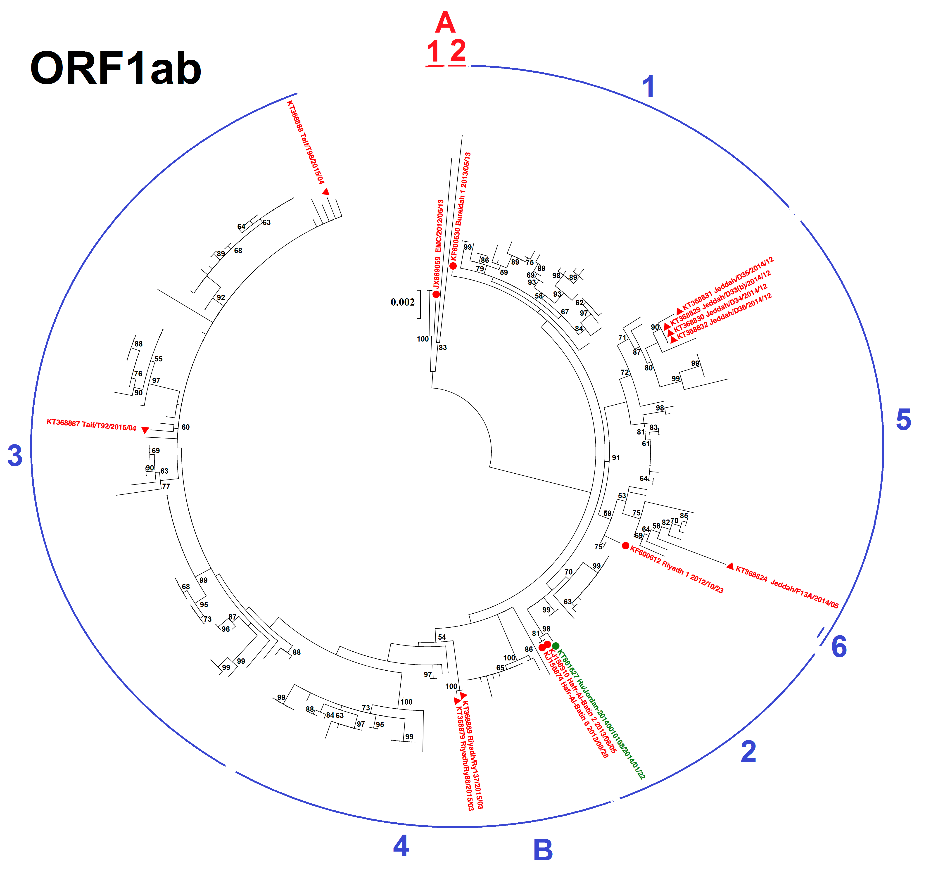


**Figure S1.** Maximum-likelihood phylogeny based on ORF1ab sequences of 219 MERS-CoV strains. GTR + G + I substitution model was selected for the ORF1ab tree. Bootstrap values in percentage are shown next to the branches. The scale bar indicates the number of nucleotide substitutions per site. Different colored letters and numbers represent different clades and lineages respectively: red letter, clade A; red number, lineages within clade A; blue letter, clade B; blue numbers, lineages within clade B. Selected MERS-CoV strains were annotated. MERS-CoVs from dromedaries are indicated in triangles. MERS-CoVs from homo sapiens are indicated in circles. The 15 MERS-CoV strains with potential recombination detected in the present study are colored: red, Saudi Arabia; green, Jordan.


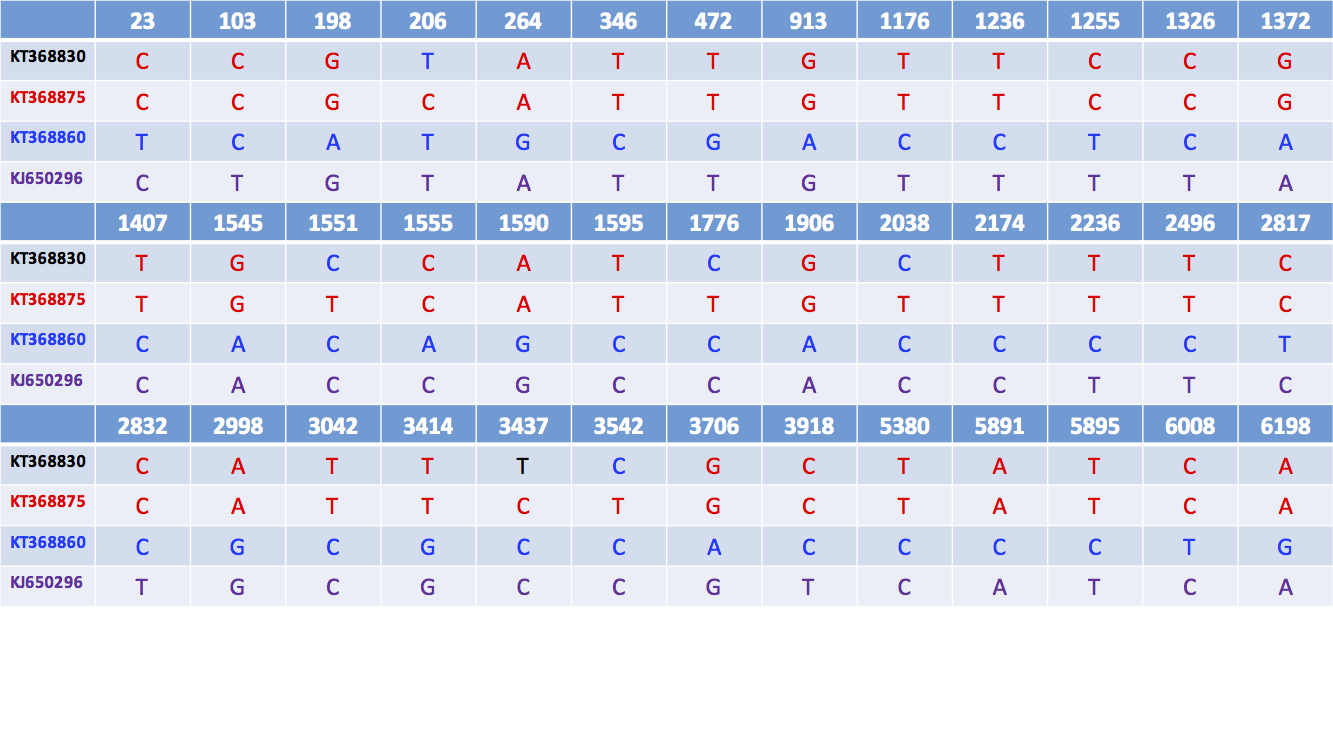


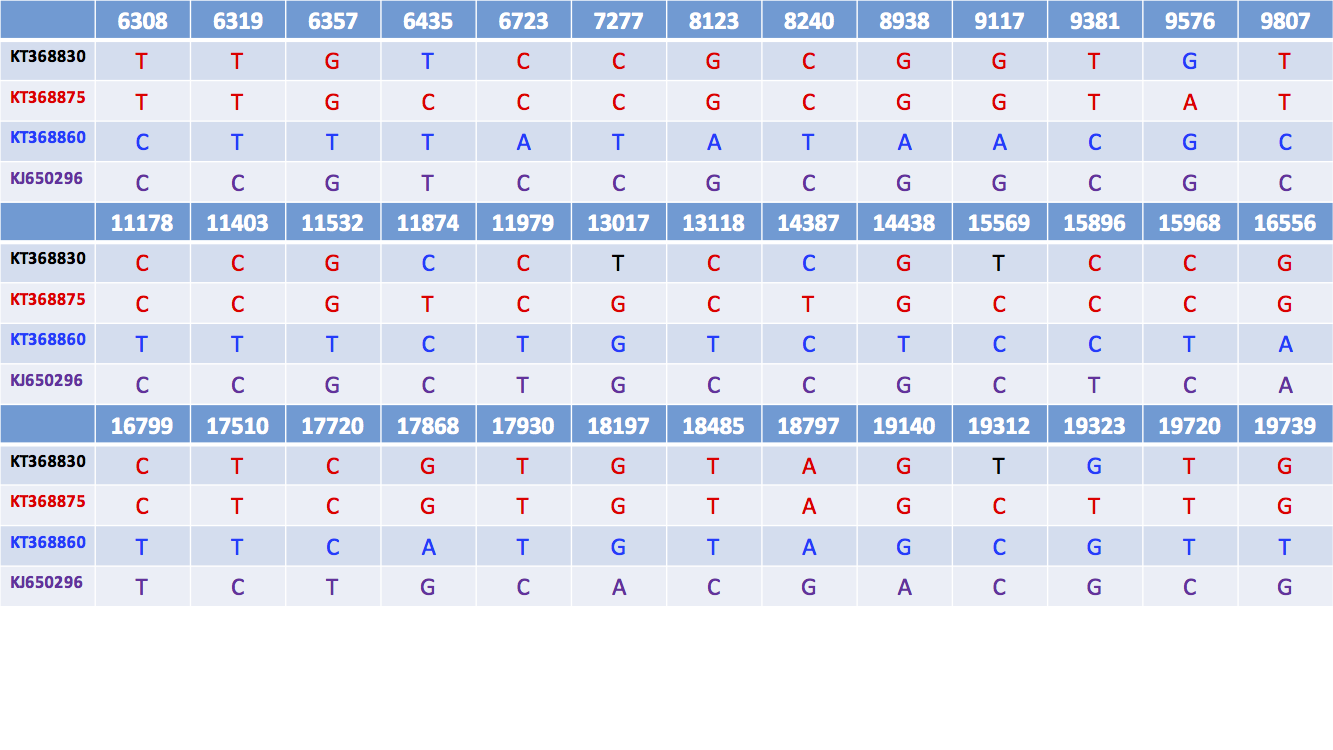


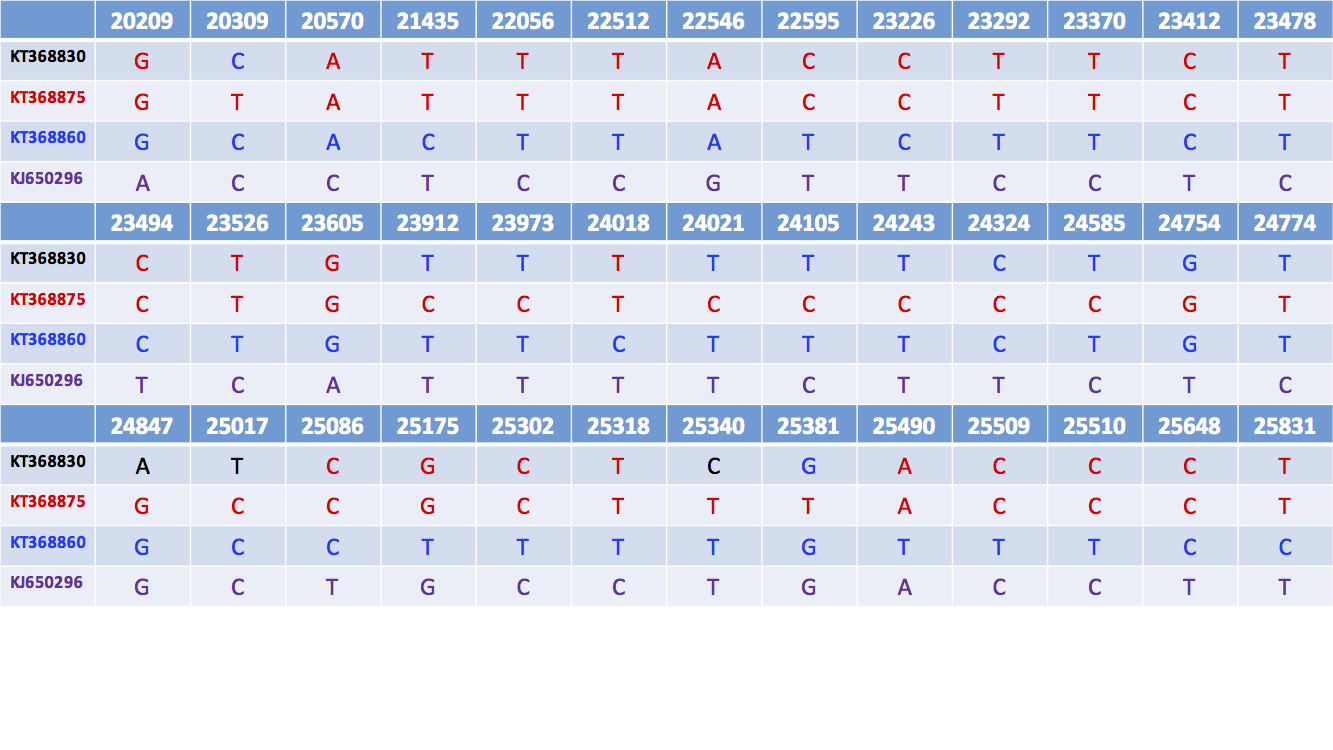


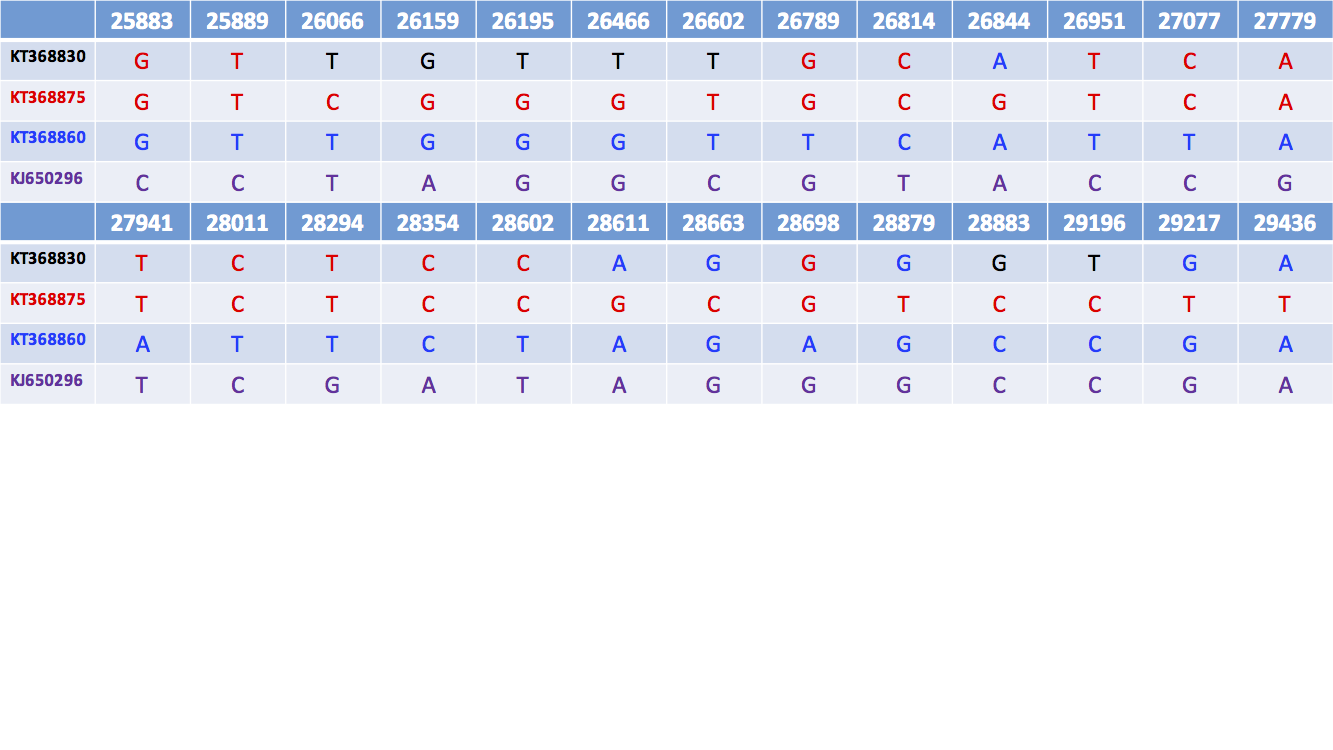


(A)


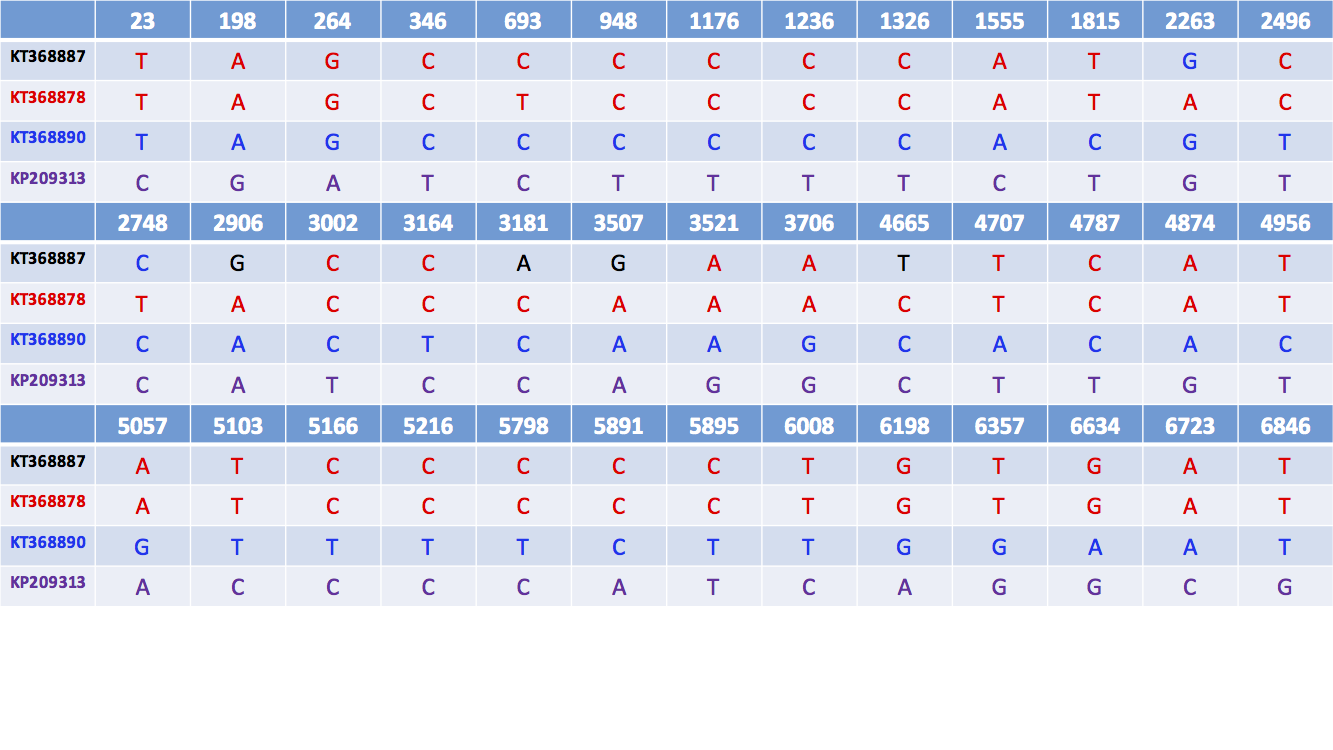


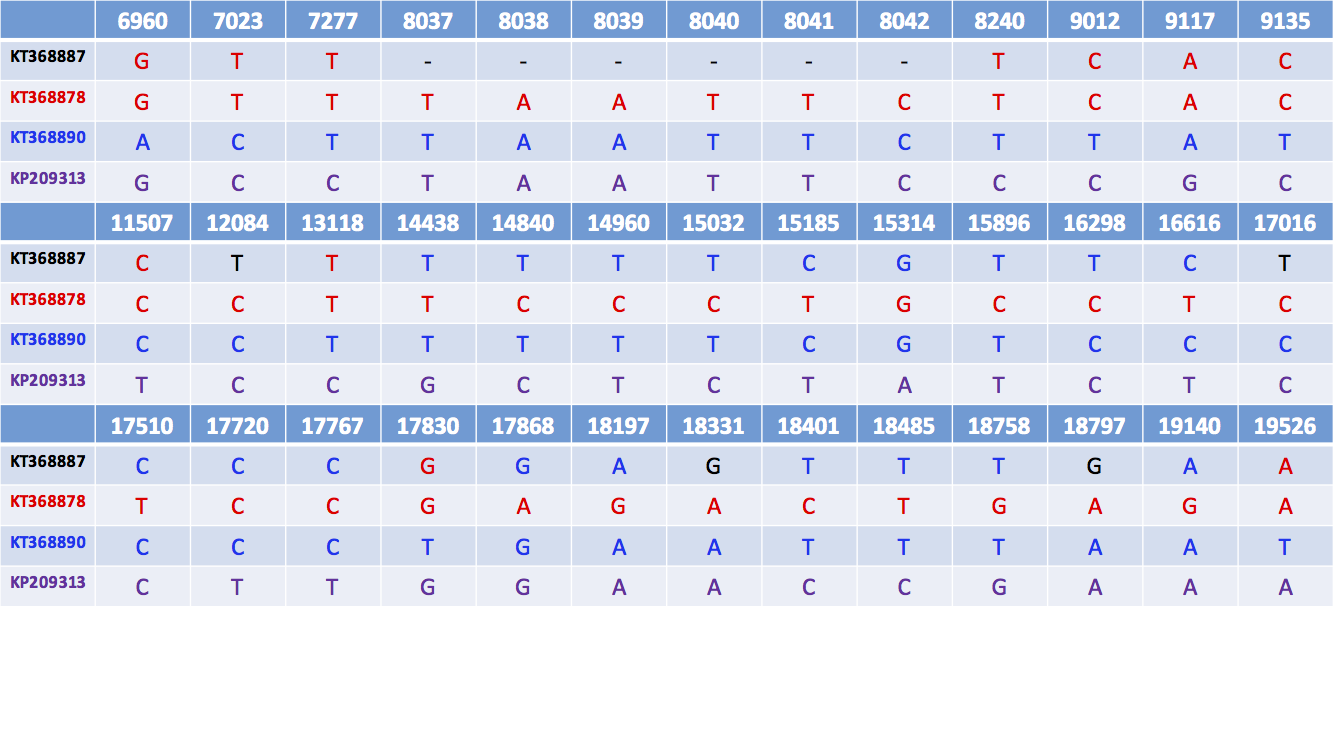


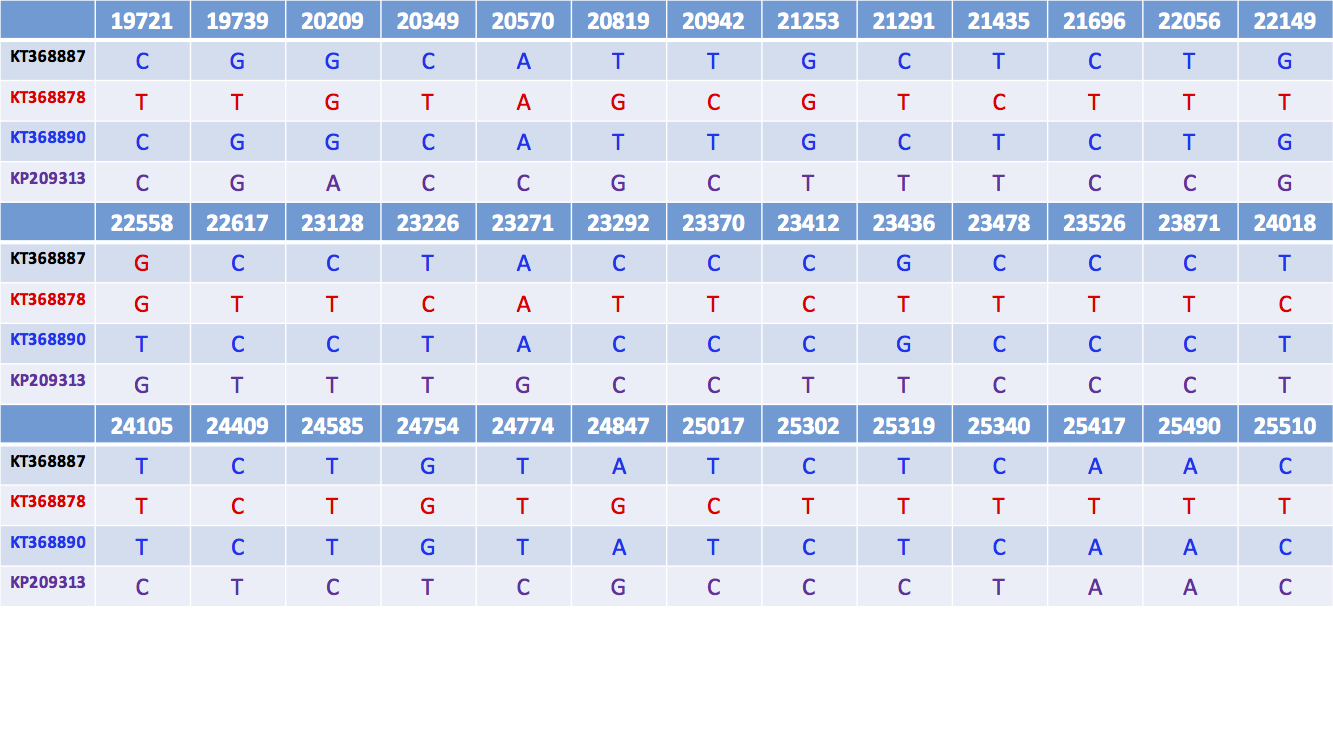


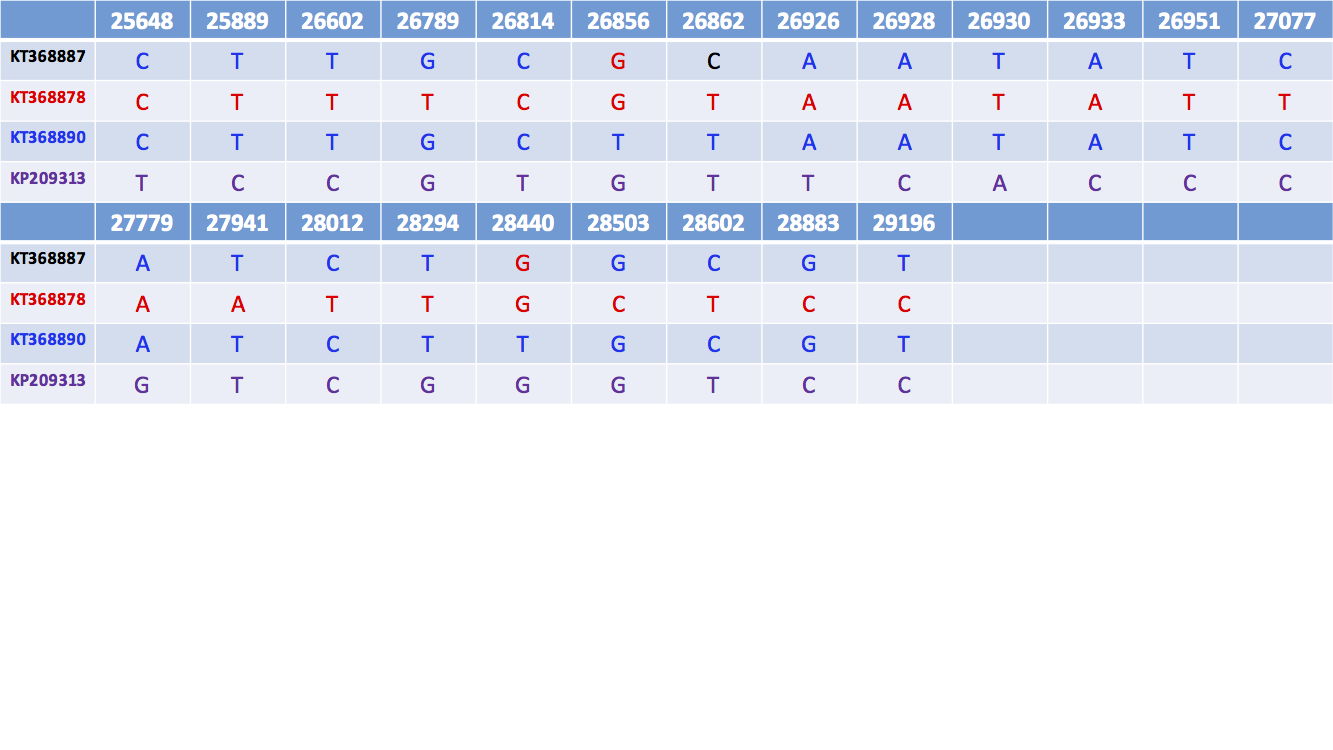


**(B)**

**Figure S2.** Multiple alignment comparison of variation sites between genome sequences. (A) KT368830 was compared with the genome sequences of a lineage B5 MERS-CoV strain (red, KT368875), a lineage B3 MERS-CoV (blue, KT368879) and a lineage B1 MERS-CoV strain (purple, KJ650926). (B) KT368887 was compared with the genome sequences of a lineage B3 MERS-CoV strain (red, KT368878), a lineage B4 MERS-CoV strain (blue, KT368890) and a lineage B1 MERS-CoV strain (purple, KP209313).
